# Assessing the resistance and bioremediation ability of selected bacterial and protozoan species to heavy metals in metal-rich industrial wastewater

**DOI:** 10.1186/1471-2180-13-28

**Published:** 2013-02-06

**Authors:** Ilunga Kamika, Maggy NB Momba

**Affiliations:** 1 Department of Environmental, Water and Earth Sciences, Faculty of Science, Tshwane University of Technology, Arcadia Campus, Private Bag X680, Pretoria, 0001, South Africa

**Keywords:** Industrial wastewater, Heavy metal, Bioremediation, Bacteria, Protozoa, Metal toxicity, Pollution, Metal-resistance

## Abstract

**Background:**

Heavy-metals exert considerable stress on the environment worldwide. This study assessed the resistance to and bioremediation of heavy-metals by selected protozoan and bacterial species in highly polluted industrial-wastewater. Specific variables (i.e. chemical oxygen demand, pH, dissolved oxygen) and the growth/die-off-rates of test organisms were measured using standard methods. Heavy-metal removals were determined in biomass and supernatant by the Inductively Couple Plasma Optical Emission Spectrometer. A parallel experiment was performed with dead microbial cells to assess the biosorption ability of test isolates.

**Results:**

The results revealed that the industrial-wastewater samples were highly polluted with heavy-metal concentrations exceeding by far the maximum limits (in mg/l) of 0.05-Co, 0.2-Ni, 0.1-Mn, 0.1-V, 0.01-Pb, 0.01-Cu, 0.1-Zn and 0.005-Cd, prescribed by the UN-FAO. Industrial-wastewater had no major effects on *Pseudomonas putida*, *Bacillus licheniformis* and *Peranema* sp. (growth rates up to 1.81, 1.45 and 1.43 d^-1^, respectively) compared to other test isolates. This was also revealed with significant COD increases (p < 0.05) in culture media inoculated with living bacterial isolates (over 100%) compared to protozoan isolates (up to 24% increase). Living *Pseudomonas putida* demonstrated the highest removal rates of heavy metals (Co-71%, Ni-51%, Mn-45%, V-83%, Pb-96%, Ti-100% and Cu-49%) followed by *Bacillus licheniformis* (Al-23% and Zn-53%) and *Peranema* sp. (Cd-42%). None of the dead cells were able to remove more than 25% of the heavy metals. Bacterial isolates contained the genes *copC*, *chrB*, *cnrA3* and *nccA* encoding the resistance to Cu, Cr, Co-Ni and Cd-Ni-Co, respectively. Protozoan isolates contained only the genes encoding Cu and Cr resistance (*copC* and *chrB* genes). *Peranema* sp. was the only protozoan isolate which had an additional resistant gene *cnrA3* encoding Co-Ni resistance.

**Conclusion:**

Significant differences (p < 0.05) observed between dead and living microbial cells for metal-removal and the presence of certain metal-resistant genes indicated that the selected microbial isolates used both passive (biosorptive) and active (bioaccumulation) mechanisms to remove heavy metals from industrial wastewater. This study advocates the use of *Peranema* sp. as a potential candidate for the bioremediation of heavy-metals in wastewater treatment, in addition to *Pseudomonas putida* and *Bacillus licheniformis*.

## Background

With rapid industrialisation all over the world, pollution of water resources is increasing drastically; South Africa is not an exception. Industrial wastewater pollution is one of the most debatable dilemmas in South Africa, where fresh water resources in global terms are scarce and extremely limited in extent. With just over 1200 m^3^ of fresh water available for each person per year for a population of around 49, 99 million, South Africa is on the threshold of the internationally used definition of water stress [1]. However, the effluent generated from domestic and industrial activities, which occupy the second position (with 14% originating from this water source, 77% from surface water and 9% from groundwater) in terms of water resources [2], currently constitutes a major source of chemical and microbial pollution of South Africa’s water sources [3].

Industrial wastewater is heavily loaded with different types of inorganic and organic pollutants, which are discharged in receiving water bodies [4]. Uncontrolled discharges of large quantities of heavy metals create not only a huge environmental and human health burden due to their high occurrence as contaminants and toxicity to all living beings [5,6], but they also increase the cost of wastewater treatment [6-8]. Toxic metal pollutants such as cadmium, nickel, lead, chromium and mercury enter the water bodies through industrial wastewater treatment [9]. Heavy metals are persistent in wastewater treatment, they are not biodegradable and their toxicity, especially in high concentrations, have become a global issue [4].

Several studies have revealed that certain microorganisms can resist the toxicity of heavy metals even at high concentrations through the acquisition of specific resistance systems such as efflux and uptake mechanisms, extracellular precipitation, etcetera [8,10,11]. Among these mechanisms, heavy metal efflux systems have been well-studied [12]. The efflux-mediated mechanism is basically a plasmid-encoded mechanism involving many operons such as *czcD, chrB, nccA* and so on, in which toxic ions enter the cell via active transport (an ATPase pump) or diffusion (a chemiosmotic ion or proton pump) [12,13]. However, this metal resistance ability is a direct response to the metal species concerned, and consequently, a particular organism may directly and/or indirectly rely on several survival strategies [11]. As a result, microorganisms are viewed as tools for the treatment of wastewater in biological processes, which have demonstrated their advantages over physico-chemical processes.

Despite the fact that several microorganisms are known to participate in the detoxification process of wastewater systems and successfully used in the production of effluent of high quality [8], the ability of protozoan species in terms of resistance to and the bioremoval of heavy metals have not been fully documented [14-16]. For decades, protozoan species have been reported as biological indicators of water quality and pollution rather than metal resistant species due to the sensitivity of certain protozoan species to the pollutants such as heavy metals [17]. As a dominant form of life on earth 1.5 billion years ago and having survived to the present day in unicellular form [18,19], protozoan species have undeniably passed through considerable challenges and evolutionary change and can also possess the potential to resist and remove heavy metals from wastewater. No specific studies have assessed the resistance of *Peranema* sp., *Trachelophyllum* sp. and *Aspidisca* sp. to highly polluted industrial wastewater systems. Due to the fact that the industrial wastewater is one of the major contributing factors of the water source pollution in South Africa, this study therefore aimed firstly at determining the effect of this source of pollution on the growth response of selected protozoan species compared to selected bacterial species, and secondly, comparing the ability of the test isolates to remove heavy metals. This study was conducted in laboratory-scale reactors which operated in batches.

## Methods

### Test organisms

In this study, three bacterial species – *Bacillus licheniformis* ATCC12759, *Brevibacillus laterosporus* ATCC64 and *Pseudomonas putida* ATCC31483 – were purchased from Quantum Biotechnologies (Strydompark Randburg, South Africa). These bacterial species have been reported for their metal tolerance or removal [20-23] and antibiotic resistance [24]. To obtain a fresh culture, an aliquot of each bacterial species was separately inoculated into a 500 ml Erlenmeyer containing 100 ml sterile nutrient broth (NB) under aseptic conditions and incubated at 30°C in a shaking incubator (Model: 353, Scientific Engineering, South Africa) with an exception of *Bacillus licheniformis* that was incubated at 50°C overnight [25]. In order to determine the cellular concentration needed for the experiment, the growth of bacterial species was measured using the spread plate method every 30 min [26].

The three protozoan species (*Aspidisca* sp.*, Trachelophyllum* sp. and *Peranema* sp.) were also obtained from the stock cultures of TUT-Water Research laboratory (South Africa). These protozoan species were previously isolated from wastewater mixed liquors collected from the aeration tanks of the Daspoort wastewater treatment plant (Pretoria, South Africa). They have been selected due to their ability to remove nitrate and phosphorus in modified mixed liquor batch reactors [27] and their moderate tolerance to nickel and vanadium [21,22]. The preparation of these protozoan species were carried out according to the process suggested by Akpor et al. [27]. Briefly, each protozoan isolates was separately transferred from the stock culture to a 500 ml Erlenmeyer containing 100 ml of fresh media of Proteose Peptone Glucose medium (PPG) under aseptic conditions. An antibiotic (streptomycin-50 μg/ml) to prevent bacterial contamination was added, including heat-killed *Eschirichia coli*-WG4 culture as a source of nutrient. To obtain the needed protozoan concentration, the inoculated flasks were incubated at room temperature (25°C) in a dark and the cell number was determined every hour using an inverted microscope (Axiovert S100, Carl Zeiss, Germany) at × 100 to × 400 magnification.

### Sample collection and preparation of the culture medium

Industrial wastewater samples were collected between November and December 2010 from a historical dumping site in a mining area at Witbank, Mpumalanga, South Africa. Prior to use, samples were allowed to settle for 2 h and were filtered through Whatman No. 1 filter papers and their profiles in terms of chemical oxygen demand (COD), dissolved oxygen (DO), pH and heavy metals were determined. The COD concentration was measured using the closed reflux method as described in standard methods [26], while the heavy metal concentrations were determined using the Inductively Couple Plasma Optical Emission Spectrometer [ICP-OES] (Spectro Ciros CCD, Spectro Analytical Instruments, Kleve, Germany). Other parameters, such as pH and DO were analysed using a pH probe (Model: PHC101, HACH) and DO probe (Model: LDO, HACH), respectively. The industrial wastewater samples, considered as culture media, were autoclaved and cooled down at room temperature before use. In order to mimic the natural environment, no supplements were added to the industrial wastewater samples. Consequently, the presence of not less than 0.2 mg/l of nutrients (nitrate, potassium, etc.) and 2.5 mg/l carbon sources were screened in the samples using standard methods, and in case the presence of these was lower, D-glucose anhydrate (2.5 g/L), MgSO_4_.7H_2_O (0.5 g/L) and KNO_3_ (0.18 g/L) purchased from Sigma Aldrich (Cape Town, South Africa) were added as a carbon source and nutrient supplement in the mixed liquor. To check the sterility of this medium, 1 ml aliquot was plated onto the sterile bacteriological agar purchased from Sigma Aldrich (Cape Town, South Africa) and incubated at 37°C for 24 h. Only flasks containing the sterile media were considered for the next step of the experimental study.

### Determination of the growth performance and heavy metal removal efficiency of test isolates in the industrial wastewater

The laboratory batch reactors consisted of 500 ml Erlenmeyer containing 300 ml of the culture media. Separate flasks were aseptically inoculated with a fresh culture of bacterial isolates (~100 CFU/ml) or protozoan isolates (~100 Cells/ml). Nutrient broth and PPG (Sigma Aldrich, SA) were used to obtain the microbial inoculums for bacteria and protozoa, respectively. Two supplementary culture media were set up as negative and positive controls. The positive control flask contained the domestic wastewater mixed liquor free of heavy metals, but inoculated with the specific test isolate, while an uninoculated industrial wastewater sample was used as the negative control. All the inoculated flasks as well as the controls were initially shaken in a shaking incubator (100 rpm) and exposed at 30°C ± 2°C. Aliquots of 40 ml were taken every day for five days to estimate the biomass and the quantity of heavy metal removed.

The microbial estimation for bacterial species was determined using the spread plate method after dilution [26]. Briefly, 100 μl of aliquot from each sample was transferred to Mannitol Egg Yolk Polymyxin (MYP) agar (Sigma Aldrich, SA), nutrient agar (NA) (Merck, SA) and *Pseudomonas* isolation agar (PIA) (Sigma Aldrich, SA) for *Bacillus licheniformis*, *Brevibacillus laterosporus* and *Pseudomonas putida,* respectively. The plates were incubated at 50°C for *Bacillus*[25] and at 30°C for the two other bacterial isolates [28]. Protozoan density was determined by a visual count using an inverted microscope (Axiovert S100, Carl Zeiss) under × 100 to × 400 magnification. The first-order die-off rate (mortality rate) and specific growth rate of the bacterial and protozoan species were calculated using the formula as reported by Peng et al. [29] and Farrier-Pagès and Rassoulzadegan [30], respectively. The die-off rate coefficient was converted to a percentage by using the total inhibition/die-off of the colony/cell counts as the 100% die-off rate.

The physico-chemical parameters such as pH, DO and COD were determined using standard methods [26]. To check the removal of heavy metals in the industrial wastewater by test organisms, an aliquot of 30 ml of the medium was taken on a daily basis, centrifuged (4000 ×g, 4°C, 15 min) and filtered using a 0.45 μm nylon filter. The remaining heavy metal concentrations were determined from the supernatants and compared with the initial heavy metal concentrations as described above. A confirmatory test was carried out by analysing heavy metal concentrations on the harvested cells previously digested. The harvested cells were washed twice with sterile deionised water, dried at 100°C in an oven, weighed and subsequently digested with high-purity nitric acid overnight, as set out by Williams et al. [31].

### Determination of metal removal efficiency of test isolates

In order to determine whether microbial isolates were using passive or active mechanisms to remove heavy metals from the mixed liquor culture media, firstly a parallel experiment study using dead (heat-killed) microbial cells (~ 6 log CFU or Cells/ml) was carried out as reported above. Secondly, microbial isolates were screened for the presence of specific metal-resistance genes.

### Isolation of DNA of the microbial species

The high molecular weight DNA was isolated from the fresh growing cells as reported by Ozutsumi et al. [32] with slight modifications. Briefly, the cell pellets harvested by centrifuging 2 ml of the fresh growing cells at 1000 ×g for 5 min at 4°C were re-suspended in 1× TE buffer (pH 8.0). The suspension were well mixed with 10 μl of Proteinase K (100 μg/μl) and 30 μl of 10X SDS then incubated at 37°C for 1 h. 80 μl of 5M NaCl and 100 μl of 10% of hexadecyltrimethyl-ammonium bromide (CTAB) were also added and incubated again for 10 min at 65°C. To remove lipid and proteins of cell membranes, an equal volume of chloroform was added and centrifuged for 5 min at 13000 ×g. The upper layer was transferred into a new eppendorf tube and mixed with an equal volume of Phenol/Chloroform/Isoamyl alcohol (25/24/1) and centrifuged for 5 min at 13000 ×g. The upper layer was transferred in a new eppendorf tube, 0.5 volume of isopropanol was added, incubated at −20°C for 30 min and then centrifuged at 13000 ×g for 5 min to precipitate DNA. To get rid of the remaining impurities and DNA inhibitor substances revealed by the nanodrop spectrophotometer results (Nanodrop2000, Thermo Scientific, Japan), the precipitated gDNA was washed with 70% ethanol and thereafter purified using ZR Fungal/Bacterial DNA Kit (Zymo Research, USA) to obtain the ratio of 260/280 value at approximately 1.8.

### PCR amplication of purified DNA

The molecular characterisation on metal-tolerance ability of test isolates were performed by the amplification of the *copABC*, *cnrB2C2, chrB, czcD* and *nccA* genes that encode for copper-chromium-zinc-nickel-cobalt-cadmium resistance, using specific primers (Table 1). The PCR amplification of the target DNA was carried out in a thermal cycler (MJ MiniTM Personal Thermal Cycler, Biorad SA) using 200-μL PCR tubes and a reaction mixture volume of 50 μL. The reaction mixture was prepared, containing 25 μl 2 × Dream Taq™ PCR master mix (10 × Dream Taq™ buffer, 2 μM dNTP mix and 1.25 U Dream Taq™ polymerase), 2 μl of each PCR primer (10 μM) (synthesised by Inqaba Biotechnologies Industry, Pretoria, South Africa) and 2 μl of genomic DNA (50 ng/μl) and was made up 50 μl with ultra-pure nuclease-free water (19 μl). The following cycling parameters were used: denaturation of template DNA at 94°C for 2 min, followed by 30 cycles of denaturation at 94°C for 1 min, annealing of template DNA for 30 s at specific temperature (Table 1) and an extension time of 1 min at 72°C for the primers. After the last cycle the samples were kept at 72°C for 10 min to complete the synthesis of all the strands and a cooling temperature of 4°C was applied. The PCR product (10 μl) was analysed using 1% (m/v) agarose gel (Merck, SA) stained with 5% of 10 mg/ml ethidium bromide (Merck, SA) and electrophoresed to determine the product size, which was visualised under UV light in an InGenius L Gel documentation system (Syngene).

**Table 1:** Primers targeting some metal-resistance genes used in this study

**Primer name**	**Mechanism involved/metal involved**	**Sequence forward (5’-3’)**	**Sequence reverse (5’-3’)**	**Annealing temperature**	**Amplicon size (bp)**
*copA*	Sequestration and transport/Cu	TCCATACACTGGCACGGCAT	TGGATCGGGTGAGTCATCAT	54	1331
*cop*B	Sequestration and transport/Cu	TCCACGTTTGTTCACTGCTC	AGTCGGCTGTATTGCCGTAG	53	900
*cop*C	Sequestration and transport/Cu	TGTTGAACCGCACAAGTTTC	GGTAATCGGGTGGGTATCG	54	350
*cnr*C2	RND (Efflux)/Co and Ni	GAGGAAGCGCTGGATTCC	GCAATTCCATCAAAGTTGTCTTGCC	55	341
*cnr*A3	RND (Efflux)/Co and Ni	GGACATTACCAACAAGCAGG	CACAAACGTCAGCGACAG	51.5	1447
*chr*B	CHR transporter (efflux/reduction)/Cr	GTCGTTAGCTTGCCAACATC	CGGAAAGCAAGATGTCGATCG	57	450
*czc*D	Cation diffusion facilitator (efflux)/Co, Zn and Cd	TTTAGATCTTTTACCACCATGGG CGCAGGTCACTCACACGACC	TTTCAGCTGAACATCATACCCTAGTT TCCTCTGCAGCAAGCGACTTC	57	1000
*ncc*A	RND (Efflux)/Ni, Co, Cd	ACGCCGGACATCACGAACAAG	CCAGCGCACCGAGACTCATCA	57	1141

### Statistical analyses

The data were statistically analysed using the Stata computer software (version: STATA V10, STATA Corp. LP, 2009). *T*-test was used to compare the two groups (Bacteria and Protozoa). One-way analysis of variances was used to compare isolates within the groups. The tests for relationships were carried out using the Pearson correlation test and the interpretation was performed at a two-sided 95% confidence limit.

## Results

### Profile of industrial wastewater samples

Table 2 summarises the profile of the industrial wastewater effluent samples before the preparation and inoculation of the test organisms. The results indicated that the pH values ranged from 3.94 ± 0.21 to 4.16 ± 0.05 and the concentration values of DO between 5.76 ± 0.05 and 6.81 ± 0.01 mg/l. The average concentration of the COD was found to be higher than 100 mg/l. Several chemical elements were found in the industrial wastewater effluent at concentrations ranged between 0.47 and 227.89 mg/l. The concentrations of V, Mg and Al in the industrial wastewater effluent samples were greater than 100 mg/l, and those of Co, Ni, Mn, Pb, Cu, Ti, Zn and Cd did not exceed 30 mg/l. Titanium was the only element present at a much lower concentration (0.47mg/l) in the industrial wastewater effluent.

**Table 2:** The profile of the industrial wastewater effluent samples used here as culture media (n = 3)

	**Sample**	** A**	** B**	** C**	** Average**	**SA Std.**
	**pH**	4.07 ± 0.01	4.16 ± 0.05	3.94 ± 0.21	4.06 ± 0.09	5.5-9.5
**Concentration (mg/l)**	**COD**	143.49 ± 2.33	116.60 ± 5.25	138.58 ± 1.05	132.89 ± 15.21	75
	**DO**	6.81 ± 0.01	5.76 ± 0.05	6.57 ± 0.03	6.38 ± 0.03	–
	**Co**	8.16 ± 1.38	8.08 ± 2.01	10.21 ± 3.02	8.82 ± 2.14	0.05*
	**Ni**	10.15 ± 3.02	9.31 ± 10.02	14.97 ± 12.02	11.48 ± 8.35	0.2*
	**Mn**	19.2 ± 7.21	17.02 ± 6.21	20.14 ± 2.75	18.79 ± 5.39	0.1
	**Mg**	191.29 ± 3.68	180.52 ± 6.37	201.94 ± 16.31	191.25 ± 8.79	–
	**V**	103.47 ± 11.32	101.482 ± 9.65	97.13 ± 4.95	100.69 ± 8.64	0.1*
	**Pb**	0.81 ± 0.01	1.77 ± 0.03	2.02 ± 0.00	1.53 ± 0.02	0.01
	**Ti**	0.24 ± 0.00	0.24 ± 0.00	0.93 ± 0.01	0.47 ± 0.00	–
	**Cu**	5.17 ± 0.02	5.2 ± 0.01	7.33 ± 0.01	5.9 ± 0.02	0.01
	**Zn**	18.31 ± 0.21	17.71 ± 0.38	23.19 ± 0.27	19.74 ± 0.29	0.1
	**Al**	227.06 ± 19.02	225.84 ± 27.38	230.77 ± 12.09	227.89 ± 19.50	–
	**Cd**	31.06 ± 0.25	19.97 ± 1.26	21.93 ± 1.38	24.32 ± 0.96	0.005

*UN-Food and Agriculture Organization (FAO, 1985); **SA Std:** National Water Act. No 36 of 1998, South Africa.

### Growth performance of test organisms in the industrial wastewater mixed-liquor media

**Figure 1:**
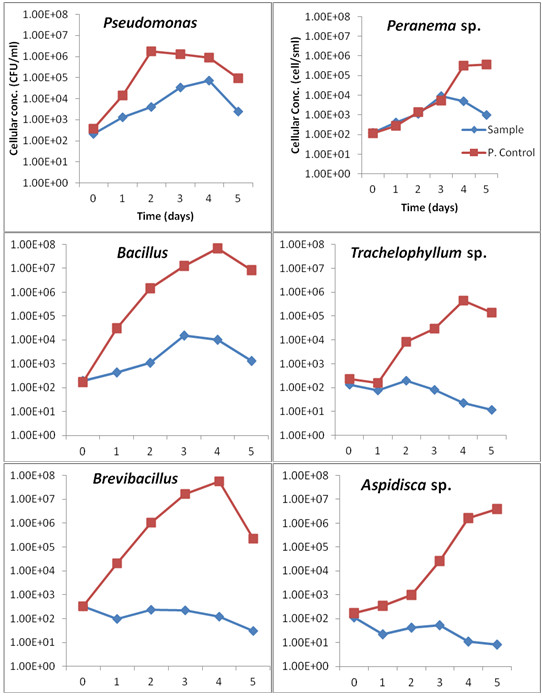
**Average growth response of bacterial and protozoan isolates exposed to industrial wastewater at pH 4 and 30 ± 2°C (n = 3) for 5 days. P. Control**: Positive control.

Figure 1 summarises the growth performance of the test organisms in the industrial wastewater mixed-liquor media during 5 d of exposure at 30°C. A general slight growth was observed in the culture media inoculated with test isolates when compared to their respective positive controls. The bacterial and protozoan counts in the industrial wastewater systems varied between 97 to 34000 CFU/ml and 8 and 9100 Cells/ml, respectively. Bacterial isolates with an exception of *Brevibacillus laterosporus* (percentage die-off rate up to 94.60%) displayed growth rates ranging between 0.5 to 1.82 d^-1^ and 0.38 and 1.45 d^-1^ for *Pseudomonas putida* and *Bacillus licheniformis*, respectively. *Pseudomonas putida* appeared to be the isolates with the highest growth rate (1.82 d^-1^) on the first day of incubation. When compared to bacterial species, protozoan isolates with exception of *Peranema* sp. revealed a gradual decrease in cell counts with *Aspidisca* sp. having a percentage die-off rate of more than 95% as the most sensitive of all isolates. *Peranema* sp. however, showed a growth rate ranging from 0.42 to 1.43 d^-1^. Statistical evidence indicated significant differences (p < 0.05) within protozoan isolates as well as within bacterial isolates. Significant differences were also noted between the two groups of microorganisms (p < 0.05).

### Variations of pH, DO and COD in the presence of test organisms

Table 3 demonstrates the variations of physicochemical parameters (pH, DO, COD) in industrial wastewater mixed-liquors inoculated with bacterial and protozoan isolates for 5 d exposure at 30°C, respectively. In general, there was a slight fluctuation in pH values over the exposure time for bacterial isolates as well as protozoan isolates in industrial wastewater samples. On the fifth day, the culture-media inoculated with *Pseudomonas putida* indicated the highest pH increase (pH 4.5 ± 0.75) when compared to all the test isolates. A gradual decrease of DO over time (Table 3) was observed, remarkably noted between the second and fourth days. For bacterial isolates, the highest DO removal of 84.4 ± 4.02% was observed in the culture media inoculated with *Pseudomonas putida*, followed by *Bacillus licheniformis* (42.73 ± 3.02%) and *Brevibacillus laterosporus* (18.61 ± 1.23%). Protozoan isolates also revealed a decrease of DO with *Peranema* sp. having the highest percentage removal of 68.83 ± 1.09%. By comparing the two groups of microorganisms, *Pseudomonas putida* had the highest DO removal followed by *Peranema* sp.

**Table 3:** Variation of physicochemical parameters of industrial wastewater culture media inoculated with microbial isolates and exposed at 30°C for 5 d (n = 3)

		**BACTERIAL ISOLATES**				
	**Initial value (in mg/l or pH unit)**	** 1d**	** 2d**	** 3d**	** 4d**	** 5d**
**pH**						
*Pseudomonas putida*	4.02 ± 0.01	4.05 ± 0.14	4.01 ± 0.03	4.06 ± 0.12	4.5 ± 0.75	4.33 ± 0.14
*Bacillus licheniformis*	4.05 ± 0.10	4.03 ± 0.21	4.04 ± 0.04	3.88 ± 0.84	4.14 ± 0.21	4.22 ± 0.02
*Brevibacillus laterosporus*	4.00 ± 0.27	4.04 ± 0.04	4.05 ± 011	3.36 ± 0.21	4.23 ± 0.07	4.36 ± 0.06
**DO removal (%)**						
*Pseudomonas putida*	6.49 ± 0.12	13.87 ± 0.24	41.27 ± 0.14	70.93 ± 4.31	84.4 ± 4.02	82.4 ± 8.24
*Bacillus licheniformis*	7.03 ± 0.17	13.1 ± 1.07	13.57 ± 1.12	13.94 ± 1.21	25.51 ± 3.21	42.73 ± 3.02
*Brevibacillus laterosporus*	6.74 ± 0.08	12.33 ± 1.28	15.35 ± 0.12	17.93 ± 0.21	38.21 ± 1.37	39.61 ± 1.23
**COD increase (%)**						
*Pseudomonas*	143.25 ± 7.12	19.56 ± 2.14	87.25 ± 7.95	159.23 ± 10.2	170.73 ± 5.18	175.86 ± 4.12
*Bacillus*	162.45 ± 10.25	29.23 ± 5.12	69.55 ± 6.89	129.28 ± 12.0	136.21 ± 1.32	142.14 ± 1.2
*Brevibacillus*	197.58 ± 9.23	7.25 ± 3.14	39.22 ± 8.14	51.08 ± 9.21	64.32 ± 2.9	68.33 ± 3.58
**PROTOZOAN ISOLATES**						
**pH**						
*Peranema* sp.	4.04 ± 0.02	3.94 ± 0.01	4.05 ± 0.05	4.06 ± 0.02	3.85 ± 0.09	3.78 ± 0.21
*Trachelophyllum* sp.	3.95 ± 0.12	3.93 ± 0.04	4.01 ± 0.17	3.96 ± 0.10	4.08 ± 0.12	3.89 ± 0.08
*Aspidisca* sp.	4.01 ± 0.07	3.94 ± 0.03	3.77 ± 0.21	4.08 ± 0.17	3.96 ± 0.26	3.88 ± 0.34
**DO removal (%)**						
*Peranema* sp.	6.43 ± 1.12	24.42 ± 2.01	33.35 ± 0.17	45.3 ± 2.07	65.22 ± 3.27	68.83 ± 1.09
*Trachelophyllum* sp.	6.74 ± 2.01	10.49 ± 0.07	18.93 ± 2.01	18.03 ± 2.01	20.33 ± 1.09	23.02 ± 2.01
*Aspidisca* sp.	5.95 ± 0.0.1	12.55 ± 0.38	11.88 ± 0.21	10.8 ± 1.09	15.25 ± 2.08	16.73 ± 2.01
**COD increase (%)**						
*Peranema* sp.	189.23 ± 9.25	7.5 ± 0.01	9.15 ± 1.02	11.25 ± 0.21	11.97 ± 0.38	12.07 ± 0.95
*Trachelophyllum* sp.	205.56 ± 6.21	16.85 ± 5.01	19.95 ± 1.97	20.12 ± 0.67	21.85 ± 0.67	23.53 ± 0.21
*Aspidisca* sp.	270.32 ± 2.21	15.25 ± 2.01	16.28 ± 1.20	20.95 ± 0.34	21.45 ± 0.21	21.43 ± 0.38

In addition, an increase in COD concentrations also occurred over time in industrial wastewater samples inoculated with test organisms. The gradual increase was noted from the second to the fifth days in industrial wastewater media inoculated with bacteria, especially with *Pseudomonas putida* and *Bacillus licheniformis,* which revealed an increase of over 100% from the third day. When inoculated with protozoan isolates, a slight increase in COD was observed with *Trachelophyllum laterosporus* showing the highest COD increase on the fifth day (Table 3).

Statistically, there were significant differences in pH variations between the industrial wastewater samples inoculated with bacteria and those inoculated with protozoa (p < 0.05) but no significant differences (p > 0.05) were noted within each group of organisms. For the DO variations, significant differences were found within protozoan isolates (p < 0.05) while bacterial isolates (p > 0.05) revealed no significant differences. Moreover, statistical analysis in terms of COD variations revealed significant differences between bacterial isolates (p < 0.05) and no significant differences within protozoan isolates (p > 0.05). However, there were also significant differences in COD variations between both groups of test organisms (p < 0.05).

### Bio-uptake of heavy metals from industrial wastewater culture media by bacterial and protozoan isolates

**Figure 2:**
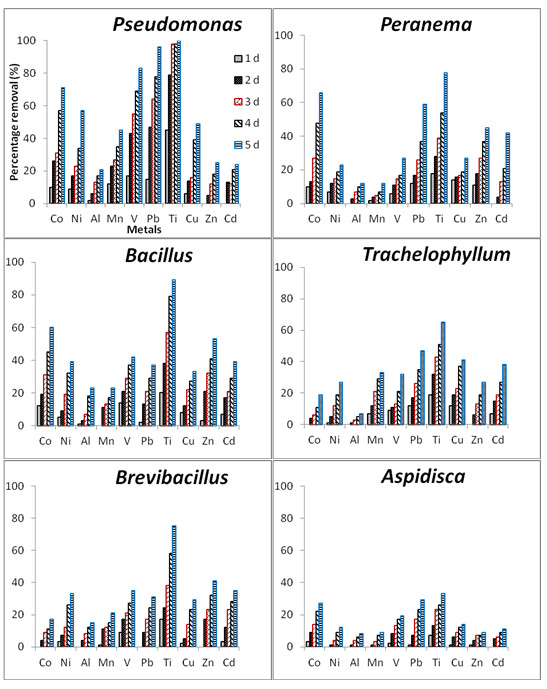
The percentage removal of heavy metals from the industrial wastewater samples by microbial isolates (n = 3).

Figure 2 illustrates the removal of heavy metal ions from industrial wastewater samples (initial concentrations of heavy metals are displayed in Table 2) by test organisms throughout the study period. In general, all test organisms exhibited a gradual increase in heavy metal removal over the exposure time. Nevertheless, higher heavy metal removal efficiencies were noted with bacterial species than with protozoan species. For bacterial isolates, with the exception of Zn, Al and Cd, *Pseudomonas putida* showed the highest removal rates for all the heavy metals (100% of Ti, 96% of Pb, 83% of V, 71% of Co, 57% of Ni, 49% of Cu and 45% of Mn), followed by *Bacillus licheniformis* with high a removal of Zn (53%), Cd (39% and Al (23%). With the exception of Ti (75%), *Brevibacillus laterosporus* indicated the lowest heavy metal removal rates (17% of Co, 33% of Ni, 21% of Mn, 35% of V, 31% of Pb,, 29% of Cu, 41% of Zn and 35% of Cd) when compared to other bacterial isolates on the fifth day of exposure (Figure 2). Among protozoan species, *Peranema* sp. exhibited the highest removal rates of Ti (78%) and Co (66%) and higher removal of Pb (59%), Zn (45%) and Cd (42%). *Trachelophyllum* sp. exhibited higher removal rates of Ni (27%), Cu (41%) and Mn (33%) compared to all the protozoan isolates. Results of this study also revealed that *Trachelophyllum* sp. had a higher removal of V (32%) compared to the other test protozoan species and that *Aspidisca* sp. was the most sensitive of all the isolates and revealed the lowest removal of all the metals.

Among the protozoan isolates, the statistical evidence revealed significant differences (p < 0.05) in heavy metal removal only for Co, Zn, Mn and Ni, but no significant differences (p > 0.05) for Cd, Pb, V, Ti, Cu and Al. Within the bacterial isolates, significant differences (p < 0.05) in heavy metal removal were found for Co, Zn, Ti, Pb, V and Mn. In addition, by comparing the two groups of test organisms, the statistical analysis showed significant differences (p < 0.05) for Co, Ti, V, Mn and Ni.

The Pearson’s correlation test was performed to establish the degree of correlations between the test organism microbial counts, pH, COD increase and the percentage removal of DO and heavy metals in the industrial wastewater samples. For bacterial isolates, the correlation test revealed a moderate correlation (0.3 < |*r*| < 0.7) between bacterial counts and all the parameters, except for the pH and the DO removal, which exhibited weak correlations (0 < |0.092, 0.188| < 0.3), and aluminum removal, which showed a strong negative correlation (|*r = −*0.971| > 0.7). By analysing the data collected for the protozoan isolates, the statistical evidence regarding the relationship between the protozoan counts and the pH, between the protozoan counts and the COD increase, as well as between the percentage removal of DO and heavy metals, revealed weak correlations (0 < |*r*| < 0.3) with the exception of Co (*r =* 0.477), Zn (*r* = 0.524), Ni (*r* = 0.332) and Al (*r* = 0.33), which indicated moderate correlations (0.3 < |*r*| < 0.7). Statistical analysis correlating microbial counts of all the microbial isolates against pH, DO removal, COD increase, and metal removal (Co, Cd, Zn, Cu, Ti, Pb, V, Mn, Ni, Al) indicated moderate correlations between mean microbial counts and all the physico-chemical parameters with the exception of DO, Cd and Cu, which revealed weak correlations.

### Determination of metal removal efficiency of test isolates

In order to determine whether microbial isolates were using passive or active mechanisms to remove heavy metals from the industrial wastewater mixed liquor culture media, firstly, the biosorption ability of test isolates was assessed by inoculating heat-killed (dead) microbial cells (approximately 6 log CFU or Cells/ml) in the culture media. Secondly, microbial isolates were screened for the presence of specific metal-tolerance genes.

**Figure 3:**
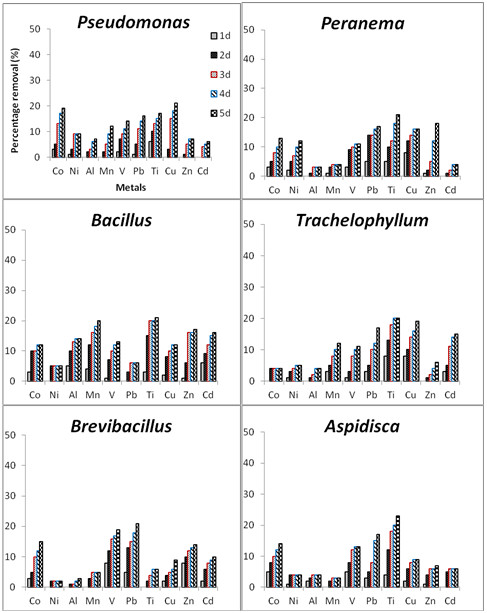
The percentage removal of heavy metals from the industrial wastewater samples by heat-killed microbial isolates (n = 3).

Figure 3 illustrates the removal of heavy metal ions from industrial wastewater samples by dead microbial cells throughout the study period. In general, a slight increase in the removal of heavy metals was observed throughout the experimental study in mixed liquor culture media. In addition, the biosorption ability of dead microbial cells in all mixed liquor culture media appeared to be exhausted after the third day of incubation. However, mixed liquor media inoculated with dead bacteria cells showed the highest removal of Co (19%), Ni (9%) and Cu (21%) for dead *Pseudomonas putida*, the highest removal of Al (14%), Mn (20%), Ti (24%), Zn (17%) and Cd (16%) for dead *Bacillus licheniformis,* and the highest removal of V (19%) and Pb (21%) for *Brevibacillus laterosporus*. Conversely, media inoculated with protozoan isolates showed the highest removal of only Ni (12%) and Zn (18%) for only dead *Peranema* sp. Statistical evidence revealed no significant difference (p > 0.05) between the heavy metal removal in the media inoculated with both dead-bacterial and dead-protozoan isolates. None of the dead-test isolates was able to remove more than 25% of the heavy metal in the culture media, with *Aspidisca* sp. indicating the highest of all (Ti-23%). This could have been due to the presence of several metals and high concentrations. However, when comparing the removal efficiency of both dead and living test isolates, statistical evidence revealed significant differences (p < 0.05).

**Figure 4:**
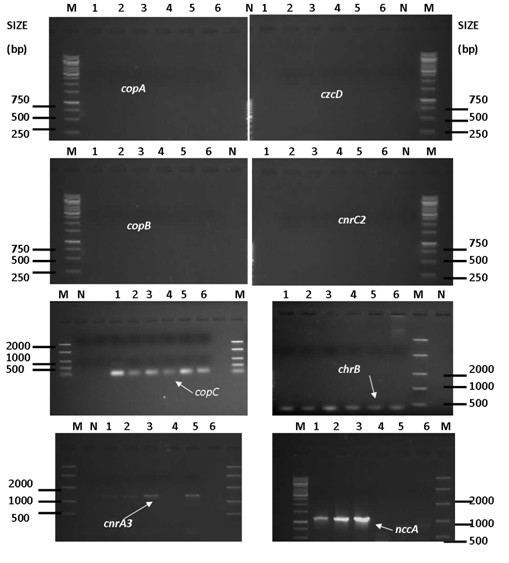
**Agarose gel electrophoresis of PCR products of total genomic DNAs with primer pair *nccA*-fwd and *nccA*-rev, primer pair *copC-*fwd and *copC*-rev, primer pair *copB-*fwd and *copB*-rev, primer pair *czcD*-fwd and *czcD*-rev, primer pair *cnrA3*-fwd and *cnrA3*-rev and primer pair *chrB-*fwd and *chrB*-rev.** Lanes: M: DNA ladder (Marker), N: Negative (No template DNA), 1 to 6, amplified PCR product of: *Pseudomonas putida* (1), *Bacillus licheniformis* (2), *Brevibacillus laterosporus* (3), *Trachelophyllum* sp. (4), *Peranema* sp. (5) and *Aspidisca* sp. (6).

To evaluate the resistance ability of the microbial isolates and whether the heavy-metal removal ability of test isolates is active, the genomic DNA was amplified with specific genes such as *copA*, *copB* and *copC* (Cu-resistance), *nccA* (Ni, Co, Cd-resistance), *cnrA3* and *cnrC*2 (Ni and Co-resistance), *chrB* (Cr-resistance) and *czcD* (Co, Zn, Cd-resistance) using the conventional PCR (Figure 4). Of all the genes targeted in the gDNA of microbial isolates, *nccA*, *cnrA3, chrB* and *copC* were the only genes to show positive amplification. For bacterial isolates (*Pseudomonas putida*, *Bacillus licheniformis* and *Brevibacillus laterosporus*), amplified products of approximately 400 bp, 450 bp, 1141 bp and 1447 bp revealing the presence of *copC* (Cu sequestration and transport), *chrB*, *nccA* and *cnrA3* genes were reproductively detected, whereas, the metal-resistant genes such as *copA*, *copB*, *cnrC2* and *czcD* were not found. However, for protozoan isolates (*Peranema* sp., *Trachelophyllum* sp. and *Aspidisca* sp.), amplified products of approximately 400 bp, 450 bp and 1447 bp revealing the presence of *copC*, *chrB* and *cnrA3* genes were found. *Peranema* sp. was the only protozoan isolate with the gene *cnrA3* (RND (Efflux)). None of the protozoan isolates revealed the presence of *copA*, *copB*, *cnrC2*, *czcD* and *nccA.*

## Discussion

The accumulation of the heavy metals in wastewater depends on many local factors, such as the type of industries in the region, way of life and awareness of the impact on the environment through the careless disposal of wastes [23,33]. Being a country with extensive industrialisation, water pollution by metal ions has emerged as one of the serious challenges currently faced by water service authorities in South Africa. Hence, this study focused on the chemical characteristics of South African industrial wastewater samples collected from one mining area at Witbank, Mpumalanga, and assessed their effect on the growth of selected bacterial and protozoan species that are among the dynamic population of wastewater and reported to be tolerant to heavy metals [21,34,35].

The finding of the present study revealed that the industrial wastewater had COD concentrations above the South African permissible limit of 75 mg/l. The pH, Mn, Pb, Cu, Zn and Cd values were also found to be beyond the South African permissible limits of 5.5 to 9.5, 0.1 mg/l, 0.01 mg/l, 0.01 mg/l, 0.1 mg/l and 0.005 mg/l, respectively. Although previous reports revealed that metals such as Co, Ni, V, Ti, Al are also toxic when present in high concentrations [4,36], no existing limits for industrial effluent discharge of these metals were found in the South African National Act of 1998 [37]. For this study, the limits set by the UN-Food and Agriculture Organization [38] and the South African National Standards (SANS, 241) for drinking water [39] were considered for these metals. Results indicated that these metals (Co, Ni, V) were present in industrial wastewater at concentrations higher than the UN-FAO permissible limits of 0.05 mg/l, 0.2 mg/l, 0.1 mg/l, respectively [38] and also at concentrations higher than the maximum limits of 1.00 mg/l, 0.35 mg/l and 0.5 mg/l, set by SANS 241, respectively. Furthermore, Al concentrations in industrial wastewaters exceeded the national standard limit of 0.5 mg/l; however, none of the regulations [37-39] has established the limit of Ti in the industrial wastewater effluent.

Although the toxicity of heavy metals to both bacteria and protozoa, previous studies reported that some microorganisms can develop detoxifying mechanisms even in water containing high concentrations of heavy metals [6,12,16]. As a result, they are used for the bioremediation of heavy metals in polluted wastewater. Intensive studies have been carried out with bacteria and their role in the bioremediation of heavy metals [6,33], whereas, few studies report on the role of protozoan species in the bioremediation of heavy metals in polluted wastewater [14,40]. The present study compared the effect of heavy metals from industrial wastewater on the growth performance of protozoan species (*Peranema* sp., *Trachelophyllum* sp. and *Aspidisca* sp.) to those of bacterial species (*Bacillus licheniformis*, *Pseudomonas putida* and *Brevibacillus laterosporu*s); they also assessed their uptake ability of heavy metals from the highly polluted industrial wastewater.

The results revealed that the exposure of both groups of test organisms in highly polluted industrial wastewater containing various heavy metals (Table 2) resulted in the inhibition of some bacterial and protozoan growth on the first day of incubation, followed by a recovery of bacterial as well as protozoan isolates between the second and third days of incubation. However, *Pseudomonas putida*, *Bacillus licheniformis* and *Peranema* sp. were able to tolerate the co-occurrence of several metals in the culture media and did not show any growth inhibition up to the fourth, third and third day of incubation, respectively (Figure 1). For *Brevibacillus laterosporus*, *Trachelophyllum* sp. and *Aspidisca* sp., the inhibition and slow growth response occurred after the second day of incubation, which could be due to the antimicrobial/toxicity effects of heavy metals as reported by Kamika and Momba [21]. As the tolerance and bioaccumulation of heavy metals by microorganisms depends on the microbial species, the culture media, the number of cells, the type of heavy metal and the presence of other metals in the samples [41], this study revealed that the industrial wastewater did not exert any major effect on the growth of *Pseudomonas putida* when compared to other bacterial isolates. Moreover, no major effect was found in the media innoculated with *Peranema* sp., which appeared to be the most tolerant protozoan isolate and the second most tolerant isolate when compared to bacterial isolates. The results of the present study are in agreement with Nilsson [42], who reported that heavy metals can affect the survival of microbial isolates in many ways such as the reduction of food uptake, growth inhibition, and reduction in the rate of endocytosis, which may influence their survival. A study conducted by Cabrera et al. [43] reported that at high concentrations, metals could slow microbial population growth. Moreover, the toxicity of these heavy metals on aerobic microorganisms can also affect the consumption of dissolved oxygen [44]. Shuttleworth and Unz [45], when investigating the effects of several heavy metals on the growth of axenic filamentous bacteria (*Thiothrix*, type 021N and type 1701), found that these organisms could grow in the presence of single toxic metals (Ca, Cu, Ni and Zn); but when mixed together, the latter appeared to act synergistically in suppressing the development of *Thiothrix* strain A1. Contrary to this, Ni^2+^ at concentrations of 10/20 mg/l was reported to stimulate the growth of *Pseudomonas putida*, *Bacillus licheniformis* and *Peranema* sp. in a modified mixed liquor medium [21]. Conversely, in the present study, the stimulating action of Ni^2+^ was not evident at similar concentrations, which could have been inhibited by the presence of other heavy metals in the industrial wastewater. Besides the pH level, the slow growth/inhibition of the test isolates might also be due to the complexity of the culture media in terms the presence of toxic ions.

Despite slight microbial growth or growth inhibition observed with regards to certain test isolates, the removal/bioaccumulation of heavy metals (Ni, Mn, Mg, V, Pb, Cu, Zn, Al, Cd) occurred in mixed liquor culture media (Figure 2). Statistically significant differences (p < 0.05) observed between the removal efficiency for dead-microbial cells (Figure 3) and living ones (Figure 2) indicated that the selected isolates were also removing heavy metals from the culture media by using active mechanisms. This was confirmed by the presence of certain specific heavy metal-resistance genes in test isolates (Figure 4). Bacterial isolates (*Pseudomonas putida*, *Bacillus licheniformis* and *Brevibacillus laterosporus*) contained the genes *copC*, *chrB*, *cnrA3* and *nccA* encoding the resistance to Cu, Cr, Co-Ni and Co-Ni-Cd, respectively, but did not contain the genes *copA*, *copB*, *cnrC2* and *czcD*. However, the presence of metal-resistant genes in *Brevibacillus laterosporus* and its growth inhibition could not be explained in the present study. Furthermore, protozoan isolates (*Peranema* sp., *Trachelophyllum* sp. and *Aspidisca* sp.) contained only the genes *copC* and *chrB* encoding the resistance of Cu and Cr, respectively. An exception was found with *Peranema* sp. that contained the gene *cnrA3* encoding the resistance of Co and Ni. This is in agreement with Mohapatra [46], who reported that apart from the sensitivity of protozoa to metal toxicants, *Peranema* is one of the protozoan isolates that are generally resistant. In addition, Ruthven and Cairns [47] reported that *Peranema* could tolerate approximately 1000 mg-Pb/l. The ability of *Pseudomonas putida* observed in this study to tolerate and remove several heavy metals from polluted industrial wastewater can be explained by the findings of Canovas and co-workers [10]. These authors reported that the genome of *Pseudomonas putida* encodes an unexpected capacity to resist heavy metals and metalloids. This species in its different strains has been reported to exhibit high maximal tolerant concentrations of a large spectrum of divalent metals [35]. Contrary to the present findings, *Pseudomonas putida* has been previously reported to contain at least four Zn/Cd/Pb efflux transporters and two czc chemiosmotic transporters [11]. It has also been reported that *Bacillus licheniformis* produce extracellular polymers with great affinity for metals; these polymers are able to complex with and accumulate metals such as Fe, Ni, Cd, etcetera [23,48].

This study corroborates the findings reported elsewhere that microorganisms can use several mechanisms to simultaneously remove metals [11,20,33]. In addition, the removal efficiency of test microorganisms mostly depended on the availability and concentrations of heavy metals in industrial wastewaters. No individual isolate showed a high removal rate of all the heavy metals from the polluted industrial wastewaters (Figure 2). High removal efficiency for only certain heavy metals was also observed in the culture media inoculated with protozoan isolates such as *Peranema* sp. This situation highlights the role of each bacterial species as well as protozoan species in the bioremediation of heavy metals in wastewater systems. Moreover, in a study conducted by Clausen [20], it was reported that *Bacillus licheniformis* CC01 could remove 93% of copper, 8% of Chromium and 45% of Arsenic while *Pseudomonas putida* could remove 25% of copper from nutrient agar. Ledin and co-workers [49] revealed in their report that *Pseudomonas putida* could remove Sr (80%), Eu (97%), Zn (70%), Cd (70%) and Hg (95%) in media containing 10^-8^ M of the respective metals.

Besides the interest revealed by several scientists with regards to bacteria for the removal of heavy metals, investigations have been undertaken on certain protozoan species in the bioremediation of and tolerance or resistance to heavy metals [50-52]. Rehman et al. [51] reported that a ciliate *Stylonychia mytilus* removed Cd (91%), Hg (90%) and Zn (98%) after 96 h of incubation in the culture media containing 10 μg/ml of the respective metal ions. In another study, Rehman and co-workers [52] also revealed that *Vorticella microstoma* can tolerate Cd (22 ug/ml), Cu (22 ug/ml), Ni (17 ug/ml), and Hg (16 ug/ml) and therefore can remove 72%, 82%, 80% and 74% of the above metals, respectively. Leborans et al. [50] also stated that certain marine protozoa communities were able to accumulate from 27.02 to 504 μg-Pb/g when they were exposed to 500 and 1000 μg/l of Pb. In addition, El-Sheekh et al. [53] reported that *Nostoc muscorum* and *Anabaena subcylindrica* were able to grow in sewage and industrial wastewater effluent and removed 12.5%-81.8% Cu, 11.8%-33.7% Co, 26.4%-100% Pb and 32.7%-100% Mn.

Unlike terrestrial environments, in aquatic environments, oxygen is usually a limiting factor and can also influence the toxicity of heavy metals to aquatic life such as aerobic microorganisms [54]. As an electron acceptor, oxygen uptake by microbial isolates in industrial wastewater could be linked to the growth of aerobic microbial isolates [48]. However, during the study period, low DO removals were recorded by all test organisms with the exception of *Pseudomonas putida* and *Peranema* sp. which showed high DO removal of 84.4 ± 4.02% and 68.83 ± 1.09%, respectively (Table 2). This situation was an indication on the toxic effect of heavy metals resulting in the slow growth of test isolates in the industrial wastewater samples. This is in agreement with Slabbert and Grabow’s finding [44], who reported that the oxygen uptake of *Pseudomonas putida* was stimulated when inoculated in diluted industrial effluent but was inhibited in highly polluted industrial wastewater.

Therefore, the DO depletion during the study could be explained by the growth of the isolates and this had also an impact on the COD which increased in the media, showing a significant microbial growth to enlighten a possible excretion of extracellular polymers involved in the heavy metal resistance [23,55]. The highest COD increase (175.86%) was noted with *Pseudomonas putida*, while *Peranema* sp. appeared to have the lowest COD increase (12.07%). The results of the present study correspond to the findings of previous investigators who also reported an increase on COD when working on the removal of nutrients [27] or on the tolerance of Ni^2+^/V^5+^[21,22] by the same test protozoan species in activated sludge mixed liquor. As opposed to this, Pala and Sponza [56] reported an efficient removal of COD in activated sludge with the addition of *Pseudomonas* sp. Musa and Ahmad [57] also reported a reduction on COD of up to 94% in wastewater when using some industrial wastewater bacterial isolates.

Statistical evidence indicated strong and moderate positive correlations consecutively between growth performance and some heavy metal removal regardless of pH, COD increase and DO removal, which could be attributed to combined microbial activities such as the biosorption of metals to cell surfaces [58], release of extracellular polymeric substances during the detoxifying process of heavy metals as well as die-off of microbial cells [59]. The weak correlations between protozoan counts and other parameters could also be attributed to the inhibition of the protozoan isolates throughout the experimental study [43].

It is well known that the pH is also an important and limiting parameter in wastewater treatment systems for the growth and activity of several organisms. In bioremediation processes, acid-tolerant microorganisms are viewed as being beneficial for the treatment of highly polluted wastewater from the mines or industry [57,60]. However, by investigating the variations of pH in the polluted industrial wastewaters, which initially had a pH of approximately 4, a slight fluctuation of pH in the inoculated industrial wastewaters was observed throughout the study period (Tables 2). Although the range of pH values for several biological activities is very narrow and ranged between 6 and 9 [48], this finding revealed that all test isolates except *Aspidisca* sp. were able to grow in an aqueous solution with a pH value of approximately 4. Akpor et al. [27], however, reported an increase in the pH value in activated sludge inoculated with some selected wastewater protozoan isolates.

## Conclusions

The outcomes of the study revealed that the South African industrial wastewater samples were highly polluted with various heavy metals, which resulted in growth inhibition of test isolates, especially protozoa. However, the growth of *Pseudomonas putida*, *Bacillus licheniformis* and *Peranema* sp. were not considerably affected by the toxic effect of the metals in the culture media. The efficiency of bacteria and protozoa in removing heavy metals from the polluted industrial wastewater mixed-liquor were found to be significantly different (p < 0.05) for most of the heavy metals with the exception of Cd, Zn, Cu, Pb and Al. In general, bacterial isolates exhibited the highest removal rates of most of the heavy metals compared to the protozoan isolates. *Pseudomonas putida* indicated the highest removal rates of most of the heavy metals followed by *Bacillus licheniformis* with a high removal of Al (23%) and Zn (53%), and *Peranema* sp. with the highest removal of Cd (42%). The study revealed that the selected bacterial species are resistant to Cu, Cr, Cd, Co, Ni (*cop*C, *chrB*, *cnrA3* and *nccA*) while the protozoan species were resistant to only Cu, Cr, Co and Ni (*cop*C, *chr*, *cnrA3*) with *Peranema* sp. being the only protozoan species able to resist Co and Ni. Moreover, the removal efficiency of test isolates was revealed, possibly due to biosorptive (passive) uptake and bioaccumulation (active uptake). Similar to the bacterial species (*Pseudomonas putida* and *Bacillus licheniformis*), *Peranema* sp. (protozoan species) has a potential application for the bioremediation of heavy metals from domestic and industrial wastewater with moderate concentrations of heavy metals. This study is one of the rare studies screening the effects of complex media containing heavy metals on members of two different kingdoms and also screening their heavy-metal removal ability. Further studies could be carried out with regards to these protozoan species, especially *Peranema* sp., in order to establish the mechanisms used to accumulate and detoxify heavy metals.

## Authors’ contributions

Conceived and designed the experiments: MNBM. Contributed reagents/materials/analysis tools: MNBM IK. Analysed the data and wrote the paper: IK. Critically reviewed the manuscript: MNBM. Both authors read and approved the final manuscript.

## References

[B1] Savenije HHG Van der Zaag P Conceptual framework for the management of shared river basins; with special reference to the SADC and EU Water Policy 2000 2 9 45 10.1016/S1366-7017(99)00021-5

[B2] Van Vuuren L The state of water in South Africa – Are we heading for a crisis? The Water Wheel 2009 8 5 31 33

[B3] Momba MNB Sibewu M Survival of somatic and F-RNA coliphages in treated wastewater effluents and their impact on viral quality of the receiving water bodies in the Eastern Cape Province J Biol Sci 2009 9 7 648 654 10.3923/jbs.2009.648.654

[B4] Jern WNG Industrial wastewater treatment 2006 Singapore: Imperial College Press

[B5] Diels L Van der Lelie N Bastiaens L New development in treatment of heavy metal contaminated soils Rev Environ Sci Biotechnol 2002 1 75 82 10.1023/A:1015188708612

[B6] Gikas P Single and combined effects of nickel (Ni(II)) and cobalt (Co(II)) ions on activated sludge and on other aerobic microorganisms: a review J Hazard Mater 2008 159 2–3 187 203 18394791 10.1016/j.jhazmat.2008.02.048

[B7] Fatta-Kassinos D Kalavrouziotis IK Koukoulakis PH Vasquez MI The risks associated with wastewater reuse and xenobiotics in the agroecological environment Sci Total Environ 2011 408 19 3555 3563 20435343 10.1016/j.scitotenv.2010.03.036

[B8] Madoni P Davoli D Gorbi G Vescovi L Toxic effect of heavy metals on the activated sludge protozoan community Water Res 1996 30 1 135 141 10.1016/0043-1354(95)00124-4

[B9] Adeniji A Bioremediation of arsenic, chromium, lead and mercury 2004 Washington: US Environmental Protection Agency, Office of Solid Waste and Emergency Response Technology Innovation Office

[B10] Canovas D Cases I De Lorenzo V Heavy metal tolerance and metal homeostasis in Pseudomonas putida as revealed by complete genome analysis Environ Microbiol 2003 5 12 1242 1256 10.1111/j.1462-2920.2003.00463.x 14641571

[B11] Leedjarv A Ivask A Virta M Interplay of different transporters in the mediation of divalent heavy metal resistance in Pseudomonas putida KT2440 J Bacteriol 1996 2680 2689 10.1128/JB.01494-07 PMC2293265 18065533

[B12] Nies DH Efflux mediated heavy metal resistance in prokaryotes FEMS Microbiol Rev 2003 27 2–3 313 339 12829273 10.1016/S0168-6445(03)00048-2

[B13] Gutiérrez JC Amaro F Martin-Gonzalez A From heavy metal-binders to biosensors: ciliate metallothioneins discussed Bioessays 2009 31 805 816 10.1002/bies.200900011 19492353

[B14] Diaz S Martin-Gonzalez A Gutierrez JC Evaluation of heavy metal acute toxicity and bioaccumulation in soil ciliated protozoa Environ Int 2006 32 6 711 717 10.1016/j.envint.2006.03.004 16650895

[B15] Martin-Gonzalez A Diaz S Borniquel S Gallego A Guitiérrez JC Cytotoxicity and bioaccumulation of heavy metals by ciliated protozoa isolated from urban wastewater treatment plants Res Microbiol 2006 157 2 108 118 10.1016/j.resmic.2005.06.005 16129584

[B16] Rajbanshi A Study on heavy metal resistant bacteria in Guheswori sewage treatment plant Our Nature 2008 6 52 57

[B17] Henebry MS Cairns J Monitoring of stream pollution using protozoan communities on artificial substrates Trans Amer Micros Soc 1980 99 2 151 160 10.2307/3225700

[B18] Weeks BS Alcamo’s microbes and society 2012 3 USA: Jones and Barlett Learning LLC

[B19] Xu J Microbial ecology in the age of genomics and metagenomics: concepts, tools, and recent advances Mol Ecol 2006 15 1713 1731 10.1111/j.1365-294X.2006.02882.x 16689892

[B20] Clausen C Isolating metal-tolerant bacteria capable of removing copper, chromium, and arsenic from treated wood Waste Manag Res 2000 18 264 268

[B21] Kamika I Momba MNB Comparing the tolerance limits of selected bacterial and protozoan species to nickel in wastewater systems Sci Total Environ 2011 440 172 181 22014510 10.1016/j.scitotenv.2011.09.060

[B22] Kamika I Momba MNB Comparing the tolerance limits of selected bacterial and protozoan species to vanadium in wastewater systems Water Air Soil Pollut 2012 223 5 2525 2539 10.1007/s11270-011-1045-9

[B23] Shirdam R Khanafari A Tabatabaee A Cadmium, nickel and vanadium accumulation by three of marine bacteria Iran J Biotechnol 2006 4 3 180 187

[B24] Choopan A Nakbud K Dawveerakul K Chawawisit K Lertcanawanichakul M Anti-methicillin resistant Staphylococcus aureus activity of Brevibacillus laterosporus strain SA14 Walailak J Sci Tech 2008 5 1 47 56

[B25] Emptage CD Knox RJ Danson MJ Hough DW Nitroreductase from Bacillus licheniformis: a stable enzyme for prodrug activation Biochem Pharmacol 2009 77 21 29 10.1016/j.bcp.2008.09.010 18840409

[B26] APHA Standard methods for the examination of water and wastewater 2001 20 Washington D.C: American Public Health Association (APHA)

[B27] Akpor OB Momba MNB Okonkwo JO Coetzee MA Nutrient removal from activated sludge mixed liquor by protozoa in a laboratory scale batch reactor Int J Environ Sci Technol 2008 5 4 463 470

[B28] Fonseca P Moreno R Rojo F Growth of Pseudomonas putida at low temperature: global transcriptomic and proteomic analyses Environ Microbiol Rep 2011 3 329 339 10.1111/j.1758-2229.2010.00229.x 23761279

[B29] Peng X Murphy T Holden NM Evaluation of the effect of temperature on the die-off rate for Cryptosporidium parvum oocycts in water, soils, and feces Appl Environ Microbiol 2008 74 23 7101 7107 10.1128/AEM.01442-08 18849452 PMC2592903

[B30] Farrier-Pagès C Rassoulzadegan F N Mineralization in planktonic protozoa Limnol Oceanogr 1994 39 2 411 419 10.4319/lo.1994.39.2.0411

[B31] Williams PN Raab A Feldmann J Meharg AA High levels of arsenic in South Central US rice grain: consequences for human dietary exposure Environ Sci Technol 2007 41 2178 2183 10.1021/es061489k 17438760

[B32] Ozutsumi Y Tajima K Takenaka A Itabashi H The effect of protozoa on the composition of rumen bacteria in cattle using 16S rRNA gene clone libraries Biosci Biotechnol Biochem 2005 69 3 499 506 10.1271/bbb.69.499 15784977

[B33] Hussein H Farag-Ibrahim S Kandeel K Moawad H Biosorption of heavy metals from waste water using Pseudomonas sp Electron J Biotechnol 2005 17 1 17 21

[B34] Brunetti G Farrag K Soler-Rovira P Ferrara M Nigro F Senesi N The effect of compost and Bacillus licheniformis on the phytoextraction of Cr, Cu, Pb and Zn by three Brassicaceae species from contaminated soils in the Apulia region, Southern Italy Geoderma 2012 170 322 330

[B35] Hu N Zhao B Key genes involved in heavy-metal resistance in Pseudomonas putida CD2 FEMS Microbiol Lett 2007 267 1 17 22 10.1111/j.1574-6968.2006.00505.x 17166231

[B36] Wang J Zhou G Chen C Yu H Wang T Ma Y Jia G Gao Y Li B Sun J Li Y Jiao F Zhao Y Chai Z Acute toxicity and biodistribution of different sized titanium dioxide particles in mice after oral administration Toxicol Lett 2007 168 2 176 185 10.1016/j.toxlet.2006.12.001 17197136

[B37] National Water Act Act No 36 of 1998 1998 South Africa: Department of Water Affairs and Forestry

[B38] FAO Water quality for agriculture 1985 Rome: Ayers ORS,Westcot DW. FAO Irrigation and Drainage Paper 29 (rev 1), Food and Agriculture Organisation

[B39] South African Bureau of Standards (SABS) South African National Standard: Drinking Water 2005 sixth SANS 241, Pretoria

[B40] Shakoori AR Rehman A Haq RU Multiple metal resistances in the ciliate protozoan, Vorticella microstoma, isolated from industrial effluents and its potential in bioremediation of toxic wastes Bull Environ Contam Toxicol 2004 72 1046 1051 15266704 10.1007/s00128-004-0349-5

[B41] Mohseni S Marzban A Sepehr S Hosseinkhani S Karkhaneh M Azimi A Investigation of some heavy metals toxicity for indigenous Acidithiobacillus ferrooxidans isolated from Sarcheshmeh copper mine Jundishapur J Microbiol 2011 4 3 159 166

[B42] Nilsson JR Effect of copper on phagocytosis in Tetrahymena Protoplasma 1981 109 359 370 10.1007/BF01287453

[B43] Cabrera G Pérez R Gomez JM Abalos A Cantero D Toxic effects of dissolved metals on Desulfovibrio vulgaris and Desulfovibrio sp. strains J Hazard Mater 2006 135 1–3 40 46 16386832 10.1016/j.jhazmat.2005.11.058

[B44] Slabbert JL Grabow WOK A rapid water toxicity screening test based on oxygen uptake of pseudomonas putida Toxicity Assess 1986 1 1 13 26 10.1002/tox.2540010103

[B45] Shuttleworth KL Unz RF Influence of metal speciation on the growth of filamentous bacteria Water Res 1991 25 10 1177 1186 10.1016/0043-1354(91)90055-U

[B46] Mohapatra PK Environmental microbiology 2008 1 New Delhi: I.K. International Publishing House

[B47] Ruthven JA Cairns J Response of fresh-water protozoan artificial communities to metals J Protozool 1973 20 127 135

[B48] Bitton G Wastewater microbiology 1999 2 Canada: Wiley-Liss

[B49] Ledin M Pedersen K Allard B Effects of pH and ionic strength on the adsorption of Cs, Sr, Eu, Zn, Cd and Hg by Pseudomonas putidia Water Air Soil Pollut 1997 93 367 381

[B50] Leborans GF Herrero OY Novillo A Toxicity and bioaccumulation of lead in marine protozoa communities Ecotoxicol Environ Saf 1998 39 172 178 10.1006/eesa.1997.1623 9570907

[B51] Rehman A Ashraf S Qazi JI Shakoori AR Uptake of lead by a ciliate, Stylonychia mytilus, isolated from industrial effluents: Potential use in bioremediation of wastewater Bull Environ Contam Toxicol 2005 75 290 296 10.1007/s00128-005-0751-7 16222500

[B52] Rehman A Shakoori FR Shakoori AR Resistance and uptake of heavy metals by Vorticella microstoma and its Potential use in industrial wastewater treatment Environ Prog Sustain Energy 2010 29 4 481 486 10.1002/ep.10450

[B53] El-Sheekh MM El-Shouny WA Osman MEH El-Gammal WE Growth and heavy metals removal efficiency of Nostoc muscorum and Anabaena subcylindrica in sewage and industrial wastewater effluents Environ Toxicol Pharmacol 2005 19 2 357 365 10.1016/j.etap.2004.09.005 21783496

[B54] Jacob U Walther H Aquatic insect larvae as indicators of limiting minimal contents of dissolved oxygen Aquatic Insects 1981 3 4 219 224 10.1080/01650428109361066

[B55] Gutierrez T Shimmield T Haidon C Black K Gree DH Emulsifying and metal ion binding activity of a glycoprotein exopolymer produced by Pseudoalteromonas sp. strain TG12 Appl Environ Microbiol 2008 74 15 4887 4876 10.1128/AEM.00316-08 PMC2519319 18552188

[B56] Pala AI Sponza DT Biological treatment of petrochemical wastewaters by Pseudomonas sp. qdded activated sludge culture Environ Technol 1996 17 7 673 685 10.1080/09593331708616434

[B57] Musa NS Ahmad WA Chemical oxygen demand reduction in industrial wastewater using locally isolated bacteria J Fund Sci 2010 6 2 88 92

[B58] Chen B Utgikar VP Harmon SM Tabak HH Bishop DF Govind R Studies on biosorption of zinc(II) and copper(II) on Desulfovibrio desulfuricans Int Biodeterior Biodegrad 2000 46 11 18 10.1016/S0964-8305(00)00054-8

[B59] Beech IB Cheung CWS Interactions of exopolymers produced by sulfate-reducing bacteria with metal ions Int Biodeterior Biodegrad 1995 35 59 72 10.1016/0964-8305(95)00082-G

[B60] Jong T Parry DL Microbial sulfate reduction under sequentially acidic conditions in an upflow anaerobic packed bed bioreactor Water Res 2006 40 13 2561 2571 10.1016/j.watres.2006.05.001 16814360

